# Guava Waste to Sustain Guava (*Psidium guajava*) Agroecosystem: Nutrient “Balance” Concepts

**DOI:** 10.3389/fpls.2016.01252

**Published:** 2016-08-29

**Authors:** Henrique A. Souza, Serge-Étienne Parent, Danilo E. Rozane, Daniel A. Amorim, Viviane C. Modesto, William Natale, Leon E. Parent

**Affiliations:** ^1^Empresa Brasileira de Pesquisa Agropecuária - Embrapa Meio-NorteTeresina, Brazil; ^2^Department of Soils and Agrifood Engineering, Université Laval, QuébecCA, Canada; ^3^Cursos de Engenharia Agronômica e Engenharia de Pesca, Universidade Estadual Paulista “Júlio de Mesquita Filho,”Registro, Brazil; ^4^Empresa de Pesquisa Agropecuária de Minas Gerais - EPAMIG OesteUberaba, Brazil; ^5^Faculdade de Engenharia, Universidade Estadual Paulista “Júlio de Mesquita Filho,”Ilha Solteira, Brazil; ^6^Departamento de Fitotecnia, Universidade Federal do CearáFortaleza, Brazil

**Keywords:** nutrient budget, isometric log ratio, nutrient balance, plant nutrition, guava

## Abstract

The Brazilian guava processing industry generates 5.5 M Mg guava waste year^−1^ that could be recycled sustainably in guava agro-ecosystems as slow-release fertilizer. Our objectives were to elaborate nutrient budgets and to diagnose soil, foliar, and fruit nutrient balances in guava orchards fertilized with guava waste. We hypothesized that (1) guava waste are balanced fertilizer sources that can sustain crop yield and soil nutrient stocks, and (2) guava agroecosystems remain productive within narrow ranges of nutrient balances. A 6-year experiment was conducted in 8-year old guava orchard applying 0–9–18–27–36 Mg ha^−1^ guava waste (dry mass basis) and the locally recommended mineral fertilization. Nutrient budgets were compiled as balance sheets. Foliar and fruit nutrient balances were computed as isometric log ratios to avoid data redundancy or resonance due to nutrient interactions and the closure to measurement unit. The N, P, and several other nutrients were applied in excess of crop removal while K was in deficit whatever the guava waste treatment. The foliar diagnostic accuracy reached 93% using isometric log ratios and knn classification, generating reliable foliar nutrient and concentration ranges at high yield level. The plant mined the soil K reserves without any significant effect on fruit yield and foliar nutrient balances involving K. High guava productivity can be reached at lower soil test K and P values than thought before. Parsimonious dosage of fresh guava waste should be supplemented with mineral K fertilizers to recycle guava waste sustainably in guava agroecosystems. Brazilian growers can benefit from this research by lowering soil test P and K threshold values to avoid over-fertilization and using fresh guava waste supplemented with mineral fertilizers, especially K. Because yield was negatively correlated with fruit acidity and Brix index, balanced plant nutrition and fertilization diagnosis will have to consider not only fruit yield targets but also fruit quality to meet requirements for guava processing.

## Introduction

Guava (*Psidium guajava*) is a tropical tree grown on Brazilian Oxisols and Ultisols and reaching high productivity 3 years after establishment (Hernandes et al., 2012). Brazil is the world leader in red guava production with 16,000 ha producing 342,000 Mg of fresh fruits annually (IBGE, 2012). The state of São Paulo accounts for 36% of total Brazilian guava production and 55% of the industrially processed production. “Paluma” is the main red guava cultivar (Natale et al., 2009). The typical guava orchard in São Paulo state is 5.6 ha in size. Crop performance for processing is measured in terms of yield, sugar content (“Brix” index) and acidity. Compared to yield, fruit sweetness and acidity may be more influenced by annual climate variations than crop management (Le Bourvellec et al., 2015). Nutrient transfers from soil to plant are influenced by climate variables and plant nutrient availability (Barber, 1995). Mineral fertilization is thus required to sustain guava yield and quality (Natale et al., 1995, 1996, 2001).

Guava processing into juice and jelly generates large amounts of waste with great potential for recycling as slow-release nitrogen fertilizers (Mantovani et al., 2004; de Souza et al., 2011). Considering that waste production is 80 kg waste Mg^−1^ of fresh fruits, the state of São Paulo generates 5.5 × 10^9^ kg of guava waste per year, most often discarded in landfills because their fertilizer value is little documented (de Souza et al., 2014a,b). In an agroecosystem approach, guava waste should be recycled to sustain plant nutrition over several guava production cycles.

In order to recycle nutrient sustainably in guava agroecosystems, guava waste additions can be guided by fertilization concepts such as “balance sheets,” “balanced” fertilization, “balanced” plant nutrition, and “balanced” nutrient ratios (Roy et al., 2006). Nutrient balance sheets report on inputs and outputs expressed in kilograms of nutrient per hectare of agricultural land (Organization for Economic Cooperation and Development, 2016). Variations in soil stocks (Kremer, 2013; Morel et al., 2014) are then interpreted using concepts such as soil nutrient buildup and maintenance to maintain soil nutrient levels close to some optimum (Dahnke and Olson, 1990), sufficiency levels of available nutrients or SLAN and basic cation saturation ratios or BCSR (McLean et al., 1983).

On the other hand, misbalanced plant nutrition can be quantified using tissue tests (Jones and Case, 1990), especially for deep-rooted plants such as fruit trees (Smith, 1985), then interpreted against isolated nutrients or ratios of nutrients at high yield level (Walworth and Sumner, 1987). Nutrient dual ratios (Walworth and Sumner, 1987) and ternary diagrams (Lagatu and Maume, 1934) have long been used to represent nutrient interactions in plant tissues (Wilkinson, 2000). However, dual ratios are asymmetrical and difficult to analyze statistically (van Kempen and Van Vliet, 2000). In addition, changing the scale of measurement for concentrations, such as dry of fresh mass basis (Walworth and Sumner, 1987) or ratios scaled on N (Ingestad, 1987), P (Güsewell, 2004), or other nutrients (Walworth and Sumner, 1987), may lead to different conclusions due to spurious correlations and sub-compositional incoherence (Aitchison, 1986). Like other compositions, soil and tissue compositions form compositional vectors constrained to some whole (Aitchison, 1986).

The SLAN, BCSR, concentration ranges and DRIS were developed before compositional data analysis techniques were found to improve the soundness and robustness of soil nutrient and plant ionome diagnoses across several agroecosystems worldwide (e.g., Parent and Dafir, 1992; Raghupathi and Bhargava, 1998; García-Hernández et al., 2004; Hernandes et al., 2012; Parent et al., 2012a,b, 2013a,b,c; Xu et al., 2015; Barłóg, 2016). Compositional vectors are made of D parts and return D-1 degrees of freedom for modeling purposes because one component can be computed by difference due to closure to measurement unit or scale (Aitchison and Greenacre, 2002). In contrast, there are D separate diagnoses for concentrations and D(D–1)/2 dual ratios and D indexes in DRIS, denying closure. Compositional nutrient diagnosis standards has been elaborated for several crops at regional scale, e.g., Rozane (2016).

Our objectives were to elaborate nutrient budgets and diagnose soil, foliar, and fruit nutrient balances in guava orchards fertilized with guava waste. We hypothesized that (1) guava waste are balanced fertilizer sources that can sustain crop yield and soil nutrient stocks under local climate conditions, and (2) guava agroecosystems are productive within narrow ranges of nutrient balances. To verify hypothesis (1), nutrient budgets were elaborated across several fertilization treatments during six guava cropping cycles where annual climate conditions and soil stocks varied. Hypothesis (2) was verified using locally derived foliar nutrient balance standards for “Paluma” guava at high yield level under the *ceteris paribus* assumption.

## Materials and methods

### Experimental site and design

Perennial fruit-bearing plants such as guava require conducting long-term experiments to document nutrient issues due to the continuous nutrient supply and removal and to internal nutrient cycling (Natale et al., 2012). The trial was conducted between 2006 and 2010 in 8 year old “Paluma” guava orchard at Vista Alegre do Alto, Sao Paulo state, 21 08′S and 48 30′W and 603 m in altitude. Climatic data were provided by a local meteorological station (Table 1). Plants were irrigated at need with artesian water at a rate of 31 L h^−1^ plant^−1^ using micro-sprinklers whenever the tensiometer installed 0.20 m below surface indicated that soil water content reached 60% of field capacity.

**Table 1:** **Average climatic conditions during the experimental period**.

**Month**	**Year**					**Mean**	**Year**					**Mean**	**Year**					**Mean**
	**2006**	**2007**	**2008**	**2009**	**2010**		**2006**	**2007**	**2008**	**2009**	**2010**		**2006**	**2007**	**2008**	**2009**	**2010**	
	**Total precipitations (mm)**						**Average temperature (°C)**											
January	237.9	430.8	355.9	239.7	287.9	310.4	19.9	20.9	19.8	19.6	20.6	20.2	30.5	28.7	28.7	29.3	30.0	29.4
February	316.6	209.6	322.0	334.9	99.2	256.5	20.0	19.9	19.2	20.1	20.4	19.9	30.1	30.4	30.7	30.7	31.6	30.7
March	193.9	128.7	182.0	172.5	180.5	171.5	20.2	19.9	17.6	19.8	19.9	19.5	30.4	31.4	29.6	30.2	30.9	30.5
April	25.9	27.2	98.3	98.6	60.7	62.1	17.2	18.8	16.4	17.0	17.0	17.3	29.2	30.6	28.8	28.8	28.9	29.3
May	13.0	71.1	32.6	21.7	19.1	31.5	12.7	14.3	12.5	15.3	13.7	13.7	26.4	26.6	26.4	27.8	26.8	26.8
June	9.8	4.9	5.5	19.4	8.9	9.7	12.8	13.5	14.1	12.0	11.9	12.9	26.8	27.4	27.2	24.7	26.8	26.6
July	5.5	65.0	0.0	19.7	3.8	18.8	12.9	12.9	12.9	14.3	13.8	13.4	28.3	26.4	28.0	27.6	28.5	27.8
August	10.1	0.0	22.2	84.8	0	23.4	14.4	14.2	15.1	14.6	13.2	14.3	27.0	27.2	30.5	28.0	30.0	28.5
September	34.7	4.3	11.9	203.4	69.2	64.7	15.8	17.5	14.7	17.9	16.9	16.6	27.7	30.8	28.4	29.2	30.9	29.4
October	110.9	60.6	47.9	68.9	125.2	82.7	18.5	19.6	19.4	18.3	17.2	18.6	30.1	33.0	31.7	30.4	30.0	31.0
November	278.3	187.3	93.7	120.2	96.7	155.2	18.8	18.6	18.9	20.8	18.5	19.1	30.3	29.6	31.5	31.7	30.5	30.7
December	343.1	120.0	212.4	257.5	187.5	224.1	20.5	19.8	19.0	20.3	20.4	20.0	29.5	30.9	30.1	29.3	30.7	30.1

Industrial guava waste consist of seeds and fruit skin and pulp. Guava waste provided each year by a nearby guava-processing plant was air-dried for 1 month from 25–30 to 5–7% moisture content at 20–30°C on a concrete floor in a shelter with sides opened for ventilation. The 50-cm layer was turned over once a week. The air-dried waste was ground mechanically (26% of dry mass in the range of 0.6–2 mm, ~61% of dry mass between 0.3 and 0.6 mm, and 13% < 0.3 mm) to facilitate handling, and stored in the shelter. Fresh residues were collected yearly from the same process and transported to the field; the 3–5 cm thick layers were exposed to relatively fast air drying (within 1–2 weeks).

The trial comprised 28 permanent plots consisting of four randomized blocks and seven treatments annually applied on the same plots during five consecutive years as mineral fertilization or guava waste. Treatments were applied manually onto soil surface without incorporation. Trees were 5 m apart on the row and row spacing was 7 m (35 m^2^ per tree) for a total of 140 trees for the 4900 m^2^ experimental orchard. There were five doses of waste (0, 9, 18, 27, and 36 Mg dry matter ha^−1^ year^−1^) applied as dried-and-ground material to facilitate manual handling, a dosage of 18 Mg dry waste-equivalent ha^−1^ year^−1^ of fresh waste as would be machine-applied in practice, and a standard treatment of locally recommended mineral fertilization. Mineral fertilizers and guava waste were spread manually each year the same day within crown projection area. The sources of mineral fertilizers were urea (45% N), ordinary superphosphate (18% P_2_O_5_, 19% Ca, 12% S), and KCl (60% K_2_O, 47.5% Cl), supplying 229 kg N ha^−1^, 12.5 kg P ha^−1^, 71.4 kg K ha^−1^, 31.5 kg Ca ha^−1^, and 19 kg S ha^−1^. Treatments started in March 2006 and continued yearly in January after soil sampling (Table 2).

**Table 2:** **Milestones of the long-term guava experiment**.

**Date**	**Fertilization**	**Soil sampling**	**Tissue sampling**	**Harvest**
March 2006	X	X		
June 2006			X	
December 2006		X		X
January 2007	X			
May 2007			X	
July–September 2007				X
November 2007			X	
December 2007		X		
January 2008	X			
February–April 2008				X
September 2008			X	
December 2008		X		
January 2009	X			
January–March 2009				X
July 2009			X	
December 2009		X		
January 2010	X			
November 2009–January 2010				X
March 2010			X	
August–October 2010				X
December 2010		X		
January 2011	X			
February 2011			X	
April–March 2011				X

### Chemical analyses

#### Waste and plant analyses

Moisture content in guava waste was determined after drying at 67 ± 2°C in a forced air oven before chemical analysis (Bataglia et al., 1983; Abreu et al., 2006). The waste was ground (<2 mm) then analyzed for macro- and micro-nutrients according to Bataglia et al. (1983). Total carbon was determined by dichromate oxidation (Abreu et al., 2006). The pH was measured in 0.01 M CaCl_2_ (1:1 volumetric ratio). The N was determined by micro-Kjeldahl including nitrate. The P and S were quantified by colorimetry and cations (K, Ca, Mg, Cu, Fe, Mn, Zn) by atomic absorption spectrophotometry after digestion in a mixture of nitric and perchloric acids. Boron was determined by colorimetry after ashing waste for 3 h in a muffle furnace at 550°C. The average nutrient composition of guava waste presented in Table 3 met the Brazilian regulation for fertilizer class “A” (Ministerio da Agricultura, 2009). The C/N ratios averaged 23.8 ± 1.4 in the dry and 23.0 ± 1.4 in the fresh waste over the 2006–2011 period. The dose of 18 Mg dry guava waste ha^−1^ provided 202–237 kg N ha^−1^ and that of fresh guava waste, 289–310 kg N ha^−1^.

**Table 3:** **Chemical analysis of the dry and fresh guava waste (mean ± standard deviation) during the 2006–2011 period**.

**Element**	**Dry guava waste**	**Fresh guava waste**	
	**2006–2011**	**2006–2011**	**Mantovani et al. (2004)**
**g kg^−1^ (DRY MASS BASIS AT 65°C)**			
C	289.0 ± 15.2	382.0 ± 5.09	355
N	12.2 ± 1.02	16.5 ± 0.58	16
P	2.20 ± 0.19	2.20 ± 0.16	2
K	2.42 ± 0.22	2.88 ± 0.08	3
Ca	0.86 ± 0.09	0.94 ± 0.13	0.5
Mg	0.96 ± 0.05	0.96 ± 0.11	0.5
S	1.26 ± 0.11	1.30 ± 0.07	−
**mg kg^−1^ (DRY MASS BASIS AT 65°C)**			
B	10.8 ± 1.84	6.7 ± 1.8	−
Cu	10.6 ± 1.14	10.5 ± 1.1	9
Fe	145.7 ± 9.5	101.7 ± 5.5	39
Mn	12.2 ± 1.3	9.7 ± 1.1	8
Zn	28.6 ± 1.8	24.7 ± 1.8	18

Twelve pairs of the first mature leaves were collected in each plot during full bloom at tree mid-height (Natale et al., 1996), then composited for tissue analysis. Foliar tissues were gently washed with distilled water, dried in a forced air oven at 65–70°C to constant weight, ground to <1 mm and analyzed for macro- and micro-nutrients as above (Bataglia et al., 1983). Fruit sugar content was measured as the “Brix” refractive index and fruit acidity was determined as 0.1 N NaOH titratable acidity (De Mello Prado et al., 2005).

#### Soil analysis

The soil was classified as dystrophic red-yellow Ultisol (EMBRAPA, 2006). Soil surface (0–0.2 m) contained 177 g clay kg^−1^, 50 g silt kg^−1^, 130 g very fine sand kg^−1^, 450 g fine sand kg^−1^, 190 g medium sand kg^−1^, and 10 g coarse sand kg^−1^. The sublayer (0.2–0.4 m) contained 260 g clay kg^−1^, 60 g silt kg^−1^, 100 g very fine sand kg^−1^, 390 g fine sand kg^−1^, 180 g medium sand kg^−1^, and 10 g coarse sand kg^−1^. Soil available nutrient levels and pH were determined yearly each December in the 0–0.20 m and 0.20–0.40 m layers where the bulk of guava roots is located (Fracaro and Pereira, 2004). In March 2006 before treatment applications, 20 soil subsamples were collected below tree crown using a Dutch sampler. Subsamples were composited, air dried, ground and sieved to <2 mm, then analyzed for pH (0.01 M CaCl_2_), organic matter content, K, Ca, Mg, and (H + Al) according to van Raij et al. (2001). The Cu, Fe, Mn, and Zn were extracted using DTPA and quantified by atomic absorption spectrophotometry. The B was extracted using the hot water method and quantified by colorimetry. The P was extracted using an exchange resin Amberlite IRA-400 (20–50 mesh), quantified by colorimetry using the ascorbic acid method, and reported as mg dm^−3^. The K, Ca and Mg were extracted by exchange resin Amberlite IRA-120 (20–50 mesh), quantified by flame photometry (K) or atomic absorption spectrophotometry (Ca, Mg), and reported as mmol_*c*_ dm^−3^. Potential acidity (H+Al) was quantified by the SMP pH buffer method (Shoemaker et al., 1961) and using the equation of Quaggio et al. (1985) to convert buffer pH into mmol_c_ (H+Al) dm^−3^. Cation exchange capacity (CEC) was calculated as the sum of cationic species. Base saturation was computed as the sum of molar concentrations of K, Ca, and Mg divided by CEC, to conduct BCSR diagnosis. Other nutrients were diagnosed in isolation using SLAN.

#### Budgeted balance sheets

Severe pruning, irrigation, and fertilization allowed fruit harvesting thrice per 2 years (Rozane et al., 2009; de Souza et al., 2012). Nutrient input by guava waste was obtained by multiplying waste dosage (Mg ha^−1^) on dry mass basis by nutrient content in kg Mg^−1^ dry mass. Nutrient removal was quantified by multiplying fruit yield in Mg fruit ha^−1^ (mean of 11% dry matter in fruit at harvest) by fruit nutrient concentration in kg nutrient Mg^−1^. Because fruit analysis was conducted at three occasions only, nutrient concentrations by treatment (four replicates times three periods, hence 12 values per treatment) were obtained averaging the ilr values then transforming them back to concentrations. Starting 90–100 days after fruit set, guava fruits were harvested 1–3 times per week during 2 months from three representative central trees per plot at proper fruit ripening, commercial size, yellow skin, and characteristic aroma for industrial processing (Salazar et al., 2006). About 60% of the crown was pruned after each harvest. Pruning residues were shredded mechanically and left on soil as mulch. Pruning residues were not analyzed hence not budgeted, assuming internal cycling. Nutrient budgets were computed between the first soil sampling in December 2006 and the last one in January 2011.

### Tissue nutrient balances

The simplest function to remove one degree of freedom without losing any information from a composition closed to 100% is the logistic variable, i.e., *log*[*x*/(1−*x*)] where one proportion “*x*” is expressed relatively to its complement “(1 − *x*).” The closing element (1 − *x*) to 100% may be defined as a filling value (*F*_*v*_) computed as the difference between the unit or scale of measurement and the analyzed nutrient, generally expressed on a dry mass basis. After statistical analysis, the *x* and (1 − *x*) centroids are recovered by back transforming the mean of the logistic variable to original units.

In many areas of natural sciences, two entities *X* and *Y* are often reduced to a single one as dual log ratio reflecting the interaction between *X* and *Y*. Using *F*_*v*_ as basis, the log of *X*/F_v_, and *Y*/*F*_*v*_ are called additive log ratios or alr (Aitchison, 1986). For three components (*X, Y*, and *F*_*v*_), there are two alrs in the compositional vector, hence removing one degree of freedom. Again, results of statistical analysis can be back-transformed to original units from three equations: the values of *log*[*X*/*F*_*v*_]and *log*[*X*/*F*_*v*_], and [*X*+*Y*+*F*_*v*_ = 100%]. However, the alrs are not independent (i.e., orthogonal) from one another: the angle of 60° between alrs makes it difficult to project them into the Euclidean space (Pawlowsky-Glahn and Egozcue, 2006).

Orthogonality is a special case of linear independence where orthogonal vectors fall perfectly at right angle to each other (Rodgers et al., 1984). Egozcue et al. (2003) were the first to derive D - 1 linearly independent variables from compositional vectors containing more than two parts. They constrained (D > 2)-parts to D - 1 isometric log ratios (ilr) as orthogonally arranged “balances” from binary partitions between two subsets of non-overlapping, strictly positive, components. The ilr data transformation can project compositions into the Euclidean space of D - 1 Cartesian coordinates. Orthogonality qualifies the isometric log ratio as the most appropriate data-transformation technique to conduct multivariate and univariate statistical analyses on compositional data (Filzmoser et al., 2009).

In this paper, we defined nutrient balances as a system of nutrient relationships using a sequential binary partition (SBP) in which nutrients labeled “+1” (group numerator) are contrasted with nutrients labeled “-1” (group denominator) in each row. A part labeled “0” is excluded. The composition is partitioned sequentially at every ordered row into two contrasts until the (+1) and (−1) subsets each contain a single part. Balances are computed as follows (Egozcue et al., 2003):

(1) $$\begin{array}{r} {ilr}_{j}=\sqrt{\frac{n_{j}^{+}n_{j}^{-}}{n_{j}^{+}+n_{j}^{-}}}ln\frac{g\left(c_{j}^{+}\right)}{g\left(c_{j}^{-}\right)}, \end{array}$$

where, in the *j*th row of the SBP, $n_{j}^{+}$ and $n_{j}^{-}$ are numbers of components at numerator (symbol +, plus) and denominator (symbol -, minus), respectively, *g*($c_{j}^{+}$) and *g*(*c*_*j*_^−^) are geometric means of components in the + and – groups, respectively. Coefficient $\sqrt{n_{j}^{+}n_{j}^{-}/\left(n_{j}^{+}+n_{j}^{-}\right)}$ is a normalization coefficient used to obtain unitary vectors on the basis. We conventionally represented balances as [-1 group denominator as components of $g\left(c_{j}^{-}\right)$ |+1 group numerator as components of *g*($c_{j}^{+}$)] because, in algebra, the minus sign is located on the left-hand side of the vector, hence the log ratio gets more negative as the weight of the geometric mean at denominator increases and conversely (Parent et al., 2013c).

Although there are *D*! × (*D* - 1)!/2^*D*^^−1^ possible sequential binary partitions (SBP) for the D - 1 balances derivable from D-parts compositions (Pawlowsky-Glahn et al., 2011), *ad hoc* SBP can be defined to facilitate interpreting meaningful balances in relation to specific issues (Parent et al., 2013c). However, because balances are orthogonal to each other, linear, or distance-based multivariate analyses return consistent results whatever the choice of the SBP. In the SBP elaborated in Table 4 the filling value between measurement unit and the sum of determined elements is contrasted with nutrients as measure of nutrient dilution or accumulation (Jarrell and Beverly, 1981), macro-nutrients and boron interact, mobile macro-nutrients are contrasted with immobile nutrients Ca and B, N, and P reflect the relationship between protein synthesis and energy requirements known as Redfield or N/P ratio, and K and Mg interact as competing cationic species (Marschner, 1986). Because cationic micronutrients depend largely on the frequency and the moment of fungicide sprays (Cu, Zn, Mn) they were excluded from the SBP to avoid this source of high variation in the compositional vector.

**Table 4:** **Sequential binary partition of six tissue nutrients and the filling value (Fv) to compute the six ilr coordinates as contrasts between orthogonally arranged subsets of components**.

**ilr**	**N**	**P**	**K**	**Ca**	**Mg**	**S**	**B**	**Fv**	**Notation [denominator | numerator]**
1	1	−1	0	0	0	0	0	0	[P |N]
2	0	0	1	0	−1	0	0	0	[Mg |K]
3	−1	−1	1	0	1	0	0	0	[N, P |K, Mg]
4	1	1	1	0	1	−1	0	0	[S |N, P, K, Mg]
5	0	0	0	1	0	0	−1	0	[B |Ca]
6	1	1	1	1	1	1	1	−1	[F_*v*_ |N, P, K, Mg, S, Ca, B]

*Subsets are assigned −1 when at denominator, and +1 when at numerator, of the log ratios*.

### Statistical analysis and nutrient diagnosis

Statistical computations were conducted in the R statistical environment (R Core Team, 2015). Compositional data transformations were computed using the R “compositions” package (van den Boogaart et al., 2014). Confidence intervals were computed at *P* = 0.05 for means comparison.

We discretized yield between low and high yielders in order to predict the yield class using a *k nearest neighbors* (knn) classification algorithm, appropriate for the Euclidean space of ilrs, as the “kknn” package launched with the R “caret” package (Kuhn, 2016). We optimized the parameters of the knn model based on the accuracy using 10-fold cross-validations through a grid of parameters, then derived sub-populations of true negative (TN), false negative (FN), true positive (TP), and false positive (FP) plant specimens. The parameters of the model were optimized with a number of 10 nearest neighbors, a distance of four in the ilr, space and a kernel set at optimum (Schliep et al., 2016). The TN specimens are considered balanced and high-yielding; FP specimens represent cases of sub-optimal nutrient concentrations, luxury consumption, or contamination; TP specimens are misbalanced due to nutrient deficiency or excess; FN specimens are balanced but show lower yield limited by other growth factors. The TN ilrs were compared to the TP ilrs using a mobile design where statistics are presented in the balance domain at fulcrums and nutrient concentrations are appreciated in buckets (Parent et al., 2013c). Accuracy was computed as (*TN*+*TP*)/(*TN*+*TP*+*FN*+*FP*). To compute critical concentration ranges, we generated a large number (100,000) of uniformly distributed values between the ranges of the confidence intervals (*P* = 0.05) about the TN ilrs. We then back-transformed each row of randomly generated ilrs to concentrations, and finally extracted the minimum and maximum univariate concentrations across the TN ilr confidence ranges.

## Results

### Nutrient budgets and soil stocks

**Figure 1:**
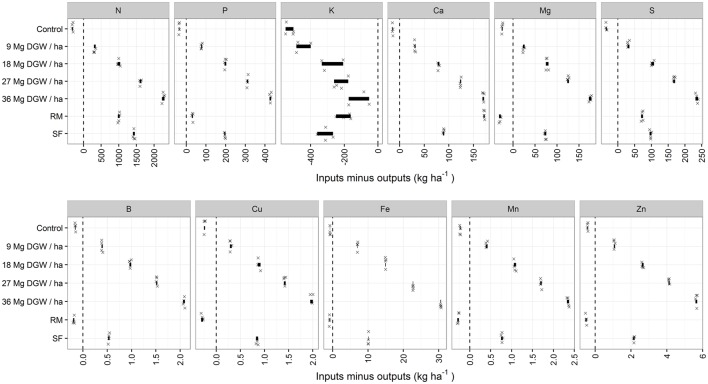
**Nutrient budgets computed by difference between nutrient inputs from fertilization and nutrient removal through harvest**. Treatments 9–36 are guava waste additions on dry mass basis (Mg DGW ha^−1^), SF represents fresh guava waste, and RM is current fertilizer recommendation as mineral fertilizers. Bold segments show dispersion of balance sheets data (*P* = 0.05 confidence intervals, four replications indicated by symbol X).

Nutrient budgets except the K were positive across guava waste treatments (Figure 1). The nutrient surpluses and K deficits increased with the dose of guava waste. As a result, nutrient use efficiency was increasingly lower for treatments exceeding 9 Mg dry guava waste ha^−1^. Ordinary superphosphate provided Ca and S in mineral fertilization in amounts comparable to or higher than guava waste, but Mg and micronutrients were in deficit with the mineral fertilization (RM) and the control, hence potentially affecting soil Mg stocks. The pH increased from 5.0 in 2006 to 5.4–6.2 in 2010 in the 0–20 cm layer but was stationary in the 0.2–0.4 m layer, indicating reduction of potential aluminum toxicity (Natale et al., 2012).

Soil analyses in the 0–0.2 and 0.2–0.4 m layers and the corresponding recommended optimum soil test values to conduct SLAN and BCSR diagnoses are presented in Table 5. In the 0–0.2 m layer, soil pH was within optimum ranges. Compared to initial conditions, soil pH increased to pH values close to 6.0, except for the mineral fertilizer treatment due likely to the acidifying action of urea. Soil test SO_4_-S increased in the mineral fertilization treatment due to gypsum (CaSO_4_.2H_2_O) in simple superphosphate. Soil test SO_4_-S did not vary across guava waste treatments despite additions of organic S. Soil tests Cu and Mn increased significantly due likely to fungicide applications while B decreased significantly in the control treatment only. Soil tests Ca and Mg appeared low, leading to base saturation near the lower limit suggested for guava (Natale et al., 1996; van Raij et al., 1997). Soil tests P and K were well below the Brazilian standards (Natale et al., 1996; van Raij et al., 1997). Soil test K was the only soil fertility index decreasing systematically across treatments in both the 0–0.2 and 0.2–0.4 m layers. The decline in soil test K confirmed the substantial mining of soil K reserves resulting from cumulated K deficits.

**Table 5:** **Soil properties in the 0–0.20 and 0.20–0.40 m soil layers under tree crown (mean ± standard error) at the onset of the experiment and gain (+) or loss (–)**.

**Property**	**Suggested targets**	**Initial soil properties**	**Dried-and-ground guava waste**					**Fresh guava waste**	**Mineral fertilization**
			**Mg dry matter ha^−1^**						
			**0**	**9**	**18**	**27**	**36**	**18**	**–**
			**Gain (+) or loss (–) compared to initial conditions (confidence intervals about mean, *P* = 0.05)**						
**0–0.2 m SOIL LAYER**									
pH (CaCl_2_)	4.5–7.0£	5.01 ± 0.03	1.15 ± 0.54	0.83 ± 0.13	1.23 ± 0.33	1.00 ± 0.22	0.40 ± 0.16	0.98 ± 0.33	0.50 ± 0.59
	5.1–5.5§								
**mg dm^−3^**									
Resin P	13–30§	10.2 ± 0.8	−1.0 ± 2.8	2.0 ± 4.1	6.8 ± 2.5	10.5 ± 9.3	12.3 ± 4.0	10.0 ± 5.0	8.8 ± 5.7
B	–	0.24 ± 0.004	−0.12 ± 0.08	−0.04 ± 0.10	−0.08 ± 0.18	−0.05 ± 0.21	−0.14 ± 0.14	−0.11 ± 0.14	−0.11 ± 0.14
Cu	–	9.8 ± 0.50	3.2 ± 2.6	3.3 ± 2.3	3.2 ± 2.1	5.1 ± 3.3	5.2 ± 4.3	5.6 ± 3.2	3.1 ± 2.0
Fe	–	17.2 ± 0.52	−4.5 ± 4.5	−4.5 ± 1.7	−5.0 ± 1.8	0.5 ± 6.1	7.0 ± 10.7	−1.3 ± 2.4	1.5 ± 1.7
Mn	–	17.5 ± 0.57	1.6 ± 5.4	7.3 ± 2.0	8.4 ± 2.9	13.9 ± 3.6	12.8 ± 3.7	13.9 ± 5.8	18.5 ± 6.6
Zn	–	0.48 ± 0.020	0.08 ± 0.38	0.13 ± 0.54	−0.03 ± 0.31	0.03 ± 0.25	0.18 ± 0.42	0.23 ± 0.15	−0.05 ± 0.16
SO_4_-S	–	2.8 ± 0.29	2.0 ± 1.4	1.3 ± 1.9	0.5 ± 1.0	1.5 ± 1.7	2.0 ± 2.6	0.3 ± 2.1	7.5 ± 4.0
**mmol_c_ dm^−3^**									
K	1.6–3.0§	2.3 ± 0.065	−1.9 ± 0.7	−1.2 ± 0.8	−1.1 ± 0.4	−1.1 ± 0.4	−1.2 ± 0.4	−1.3 ± 0.7	−1.8 ± 0.3
Ca	27§	15.3 ± 0.49	10.3 ± 11.9	7.3 ± 2.6	4.8 ± 2.8	5.8 ± 1.7	2.3 ± 3.8	3.8 ± 2.1	4.5 ± 7.6
Mg	9§	6.4 ± 0.27	3.8 ± 4.6	4.5 ± 3.9	3.5 ± 1.3	5.0 ± 0.8	3.8 ± 1.5	4.8 ± 2.5	2.8 ± 2.6
Σ bases	–	24.1 ± 0.7	12.2 ± 15.9	9.8 ± 3.6	7.2 ± 4.1	9.6 ± 2.3	4.8 ± 5.1	7.2 ± 3.7	5.5 ± 10.3
CEC	–	43.8 ± 0.7	6.4 ± 13.3	6.3 ± 4.6	3.9 ± 4.6	6.1 ± 3.0	6.1 ± 3.9	3.2 ± 3.4	7.3 ± 6.4
**%**									
Base saturation	51–70§	54.5 ± 1.65	14.8 ± 9.7	21.6 ± 13.7	13.2 ± 9.6	11.9 ± 11.0	−0.8 ± 6.5	9.3 ± 5.2	−3.4 ± 18.1
**g dm^−3^**									
OM		13.3 ± 0.2	−1.5 ± 4.8	2.0 ± 1.8	−1.3 ± 3.3	1.8 ± 1.5	−0.3 ± 1.0	1.0 ± 4.7	0.8 ± 4.3
**0.2–0.4 m SOIL LAYER**									
pH (CaCl_2_)	4.5–7.0£	5.11 ± 0.06	0.70 ± 0.35	0.45 ± 0.55	0.55 ± 0.42	0.68 ± 0.42	0.48 ± 0.51	0.58 ± 0.27	0.30 ± 0.39
	5.1–5.5§								
**mg dm^−3^**									
Resin P	13–30§	5.36 ± 0.32	−1.3 ± 2.1	−0.3 ± 2.3	1.3 ± 5.8	3.3 ± 2.5	3.0 ± 1.0	3.3 ± 4.8	1.0 ± 2.0
B	–	0.18 ± 0.018	−0.09 ± 0.14	−0.05 ± 0.07	−0.06 ± 0.14	−0.02 ± 0.19	0.10 ± 0.24	−0.08 ± 0.03	−0.05 ± 0.08
Cu	–	2.8 ± 0.30	0.65 ± 0.76	0.65 ± 0.38	0.93 ± 1.70	1.45 ± 1.80	1.70 ± 2.92	0.55 ± 1.88	0.73 ± 1.48
Fe	–	11.5 ± 0.29	−2.8 ± 1.8	−2.5 ± 1.2	−1.8 ± 0.6	−2.0 ± 1.7	−0.3 ± 1.2	−2.0 ± 1.4	0.3 ± 1.2
Mn	–	14.8 ± 0.99	7.4 ± 9.8	7.2 ± 3.6	9.0 ± 2.1	9.8 ± 2.3	15.1 ± 0.6	10.8 ± 5.4	16.3 ± 8.1
Zn	–	0.25 ± 0.015	−0.13 ± 0.06	0.10 ± 0.10	−0.05 ± 0.16	−0.05 ± 0.12	0.03 ± 0.12	−0.15 ± 0.07	−0.10 ± 0.10
SO_4_-S	–	6.7 ± 0.45	−3.3. ± 1.5	−0.5 ± 5.1	−1.5 ± 4.1	−4.0 ± 3.0	−1.3 ± 2.5	−1.3 ± 1.5	1.0 ± 4.6
**mmol_*c*_ dm^−3^**									
K	1.6–3.0§	1.8 ± 0.06	−1.2 ± 0.9	−1.0 ± 0.9	−1.2 ± 0.5	−1.3 ± 0.4	−0.9 ± 0.9	−0.9 ± 0.9	−0.9 ± 0.4
Ca	27§	13.0 ± 0.51	2.5 ± 4.2	1.0 ± 2.6	3.3 ± 5.6	1.8 ± 2.5	3.5 ± 4.5	4.0 ± 3.9	−0.5 ± 1.6
Mg	9§	5.5 ± 0.30	1.8 ± 2.7	0.8 ± 0.6	1.8 ± 2.3	1.8 ± 1.2	2.8 ± 0.6	3.0 ± 2.2	1.0 ± 2.0
Σ bases	–	20.4 ± 0.7	3.1 ± 6.0	0.8 ± 2.3	3.8 ± 5.6	2.2 ± 3.4	5.4 ± 4.6	6.1 ± 6.2	−0.4 ± 3.4
CEC	–	38.1 ± 0.8	1.6 ± 5.9	1.3 ± 6.7	3.3 ± 3.9	1.2 ± 4.4	7.9 ± 3.8	5.8 ± 7.6	−0.2 ± 4.0
**%**									
Base saturation	51–70§	53.8 ± 0.9	6.2 ± 9.4	−0.1 ± 5.7	4.6 ± 8.1	3.8 ± 9.3	2.5 ± 10.7	5.5 ± 2.2	−4.6 ± 9.5
**g dm^−3^**									
OM		8.6 ± 0.4	−0.5 ± 1.6	−0.3 ± 2.3	1.3 ± 5.8	−0.3 ± 3.2	1.0 ± 4.0	0.8 ± 4.0	0.3 ± 2.1

£ *Crane and Balerdi (2015)*.

§ *Natale et al. (1996), van Raij et al. (1997)*.

### Climate and nutrient balances impact on fruit yield and quality

**Figure 2:**
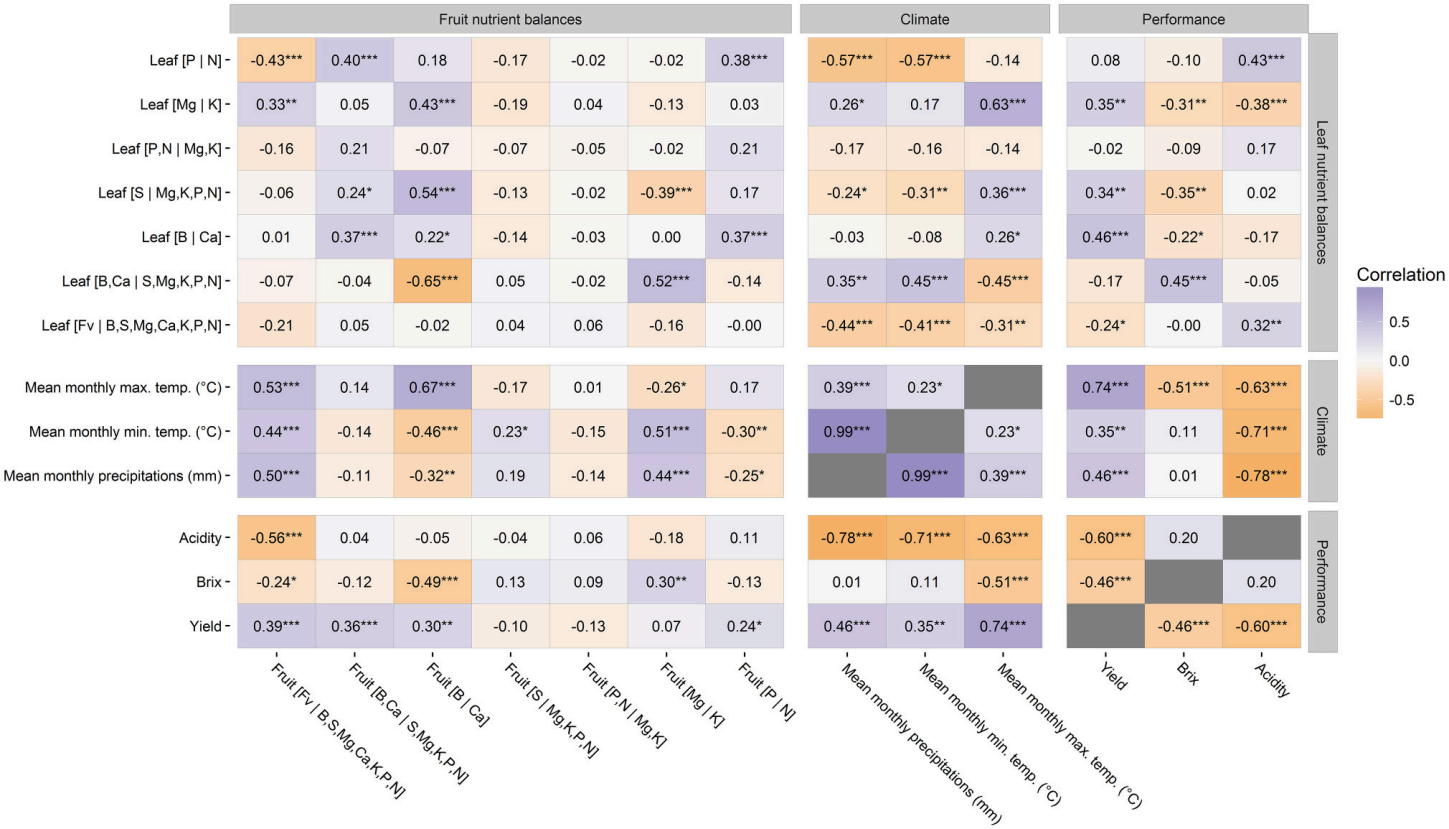
**Correlations between fruit and foliar nutrient balances, climate conditions, and crop performance**. ^*^0.01 < *p*-value ≤ 0.05; ^**^0.001 < *p*-value ≤ 0.01; ^***^ *p*-value ≤ 0.001.

Increased precipitations and the minimum and maximum monthly temperature were correlated positively with fruit yield but negatively with fruit acidity (Figure 2). Precipitations and temperature influenced both the Brix index and yield but in different directions. The Brix index significantly decreased with maximum temperature, indicating an upper temperature limit for fruit sweetness that was exceeded during the experiment. Because temperature varied little across the experimental period while total precipitations varied widely, 2009 being the wettest year and 2010, the driest, subtle variations in temperature as well as large variations in precipitations could impact on fruit yield and quality. Climate thus nurtured the ties between high fruit yields and fruit characteristics important to the processing industry.

Foliar nutrient balances were also influenced by climate conditions. Higher maximum and minimum temperatures and precipitations increased foliar nutrient accumulation vs. the filling value. The leaf [B |Ca] balance increased significantly with maximum temperature while the [P |N] balance decreased with minimum temperature and precipitations that may slow down organic N mineralization. The leaf [Mg |K] balance increased with maximum temperature, indicating a prominent role of K for osmotic adjustment to maintain higher cell turgor pressure (Wang et al., 2013). The balance between relatively immobile (B, Ca) and relatively mobile (S, Mg, K, P, N) nutrients increased with precipitations and minimum temperature. In the present experiment, maximum temperature apparently hastened convective flow of Ca and B to the leaf, hence increasing the foliar [B,Ca |S,Mg,K,P,N] balance.

Positive correlations between fruit and foliar nutrient balances were found to be significant only for the [B |Ca] and [P |N] balances, indicating proportionate supply of those nutrients to leaf and fruit between blooming and harvest and little proportionality for other nutrients. Fruit yield was correlated positively with the fruit [F_v_ |B,Ca,S,Mg,K,P,N], [P |N], [B,Ca |S,Mg,K,P,N], and [B |Ca] balances (Figure 2). In contrast, fruit acidity was correlated negatively with the fruit [F_v_ |B,Ca,S,Mg,K,P,N] balance, indicating that fruit acidity increased with lesser nutrient accumulation in the fruit. The Brix index was correlated negatively with the fruit [F_v_ |B,Ca,S,Mg,K,P,N] and [B |Ca] balances, and positively with the fruit [Mg |K] balance, indicating that fruit sweetness increased with fruit B and K concentrations in particular but with nutrient dilution in general. Although different fruit nutrient balances can lead to different levels of fruit yield and quality, plant nutrient status is most commonly diagnosed using interpretation methods that relate foliar tissue analysis to crop yield to secure the yield.

### Nutrient standards at high yield level using k nearest neighbors classification

**Figure 3:**
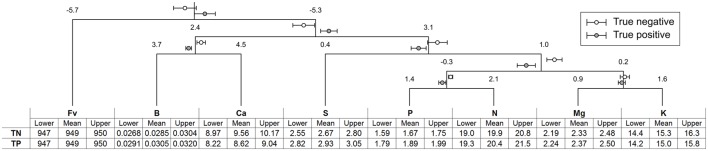
**Mobile design showing the domain of nutrient balances with confidence intervals (***P*** = 0.05) at fulcrums and the nutrient concentration domain in buckets showing minimum, mean, and maximum concentration values for true negative (TN) and true positive (TP) specimens**.

The accuracy of the partition using the k nearest neighbors (knn) classification across ilrs was 82% in cross-validation and 93% on the whole data set, with 96 true negatives, 85 true positives, 4 false negatives, and 10 false positives (negative predictive value = 0.96; positive predictive value = 0.89; specificity = 0.91; sensitivity = 0.96). Confidence intervals (*P* < 0.05) about ilrs and the corresponding minimum and maximum concentration values for TN and TP specimens are presented in the mobile design in Figure 3 where statistical analyses are conducted at fulcrums in the balance domain and nutrient concentrations are appreciated in buckets using minimum and maximum values. Despite large K deficits, the K concentration ranges and the K balances involving K did not differ between TN and TP specimens, because the soil K stocks declined markedly to support plant K nutrition. Concentration ranges for TN and TP specimens did not overlap for P and S, and nearly so for Ca and B. Apparent critical values were 1.77 g P kg^−1^, 2.81 g S kg^−1^, 9.00 g Ca kg^−1^, and 30 mg B kg^−1^. Because other ranges overlapped between TN and TP specimens, apparent critical values could not be computed for that guava agroecosystem.

## Discussion

### Nutrient budgets

Despite small annual variations in compositions between batches of guava waste, the differences between nutrient inputs and outputs remained linear (Figure 1). At maximum fruit yield of 60–61 Mg fruit ha^−1^ (de Souza et al., 2014b), all nutrients but K were found to be in excess of removal through harvest, indicating potential soil accumulation of all nutrients but K. While guava waste has been classified as a slow-released N fertilizer (de Souza et al., 2011), indicating potential residual effects of guava waste on the following crop as was the case for manure and compost (Eghball et al., 2004), there was no N shortage even at low dosage. Although guava yield response was found to be linearly related to added guava waste (de Souza et al., 2014b), a closer examination of the data showed that yield response apparently plateaued at 9 Mg ha^−1^ where added N was 101–118 kg N ha^−1^.

de Souza et al. (2011) found that 30% of total N from 9 Mg guava waste ha^−1^ was mineralized after 126 days in a laboratory incubation experiment. The first-order kinetics models returned maximum mineralizable N of 69 mg N kg^−1^ for the control without amendment and 167 mg N kg^−1^ added as 9 Mg guava waste ha^−1^. The difference was 98 mg mineralized N kg^−1^ compared to 52.2 mg total N kg^−1^ added to soil as guava waste, indicating priming effect of guava waste on soil organic matter decomposition. Indeed, organic matter decomposition is mediated by fast- and slow-growing microbial communities specialized for utilizing various sources of organic matter (Chen et al., 2014). The first-order models showed that an amount of 52.2 mg total N kg^−1^ from guava waste was mineralized after 100 days, well within the 10 months period required to complete the guava cycle. Irrigation and heavy rainfall may have further stimulated biological activity and organic matter mineralization under field conditions (Dersch and Böhm, 2001; Calderón and Jackson, 2002; Sainju et al., 2010; Condron et al., 2014).

While K is extracted in large amounts by guava fruits (Natale et al., 1994), the K deficit did not affect crop performance during the 6 years of experimentation. Indeed, fruit species can consume internal nutrient reserves to supply the demand for fruit production (Adrian et al., 2015). It is often assumed that the change in plant nutrient reserves at steady state is approximately equal to change in the pruned nutrient biomass that is left at soil surface (Tagliavini and Scandellari, 2012). The contribution of pruning residues to nutrient cycling was not quantified in this study but can be assessed from literature. Guava pruning residues may contain 1.9–8.2% ash, of which 25–52% was K, 20–37% Ca, 7–16% Mg, 5–9% P, and 3–5% S, while a guava tree returned 9–12 kg of pruning biomass after fruit harvesting (Camarena-Tello et al., 2015). Assuming concentration averages and 10.5 kg pruning residues tree^−1^, pruning residues could have contributed 152 kg K ha^−1^, 45 kg Mg ha^−1^, and 112 kg Ca ha^−1^, i.e., 3.89 mmol_c_ K dm^−3^, 3.77 mmol_c_ Mg dm^−3^, and 5.61 mmol_c_ Ca dm^−3^ to soil reserves. Pruning residues apparently sustained soil Ca and Mg during the experimental period but not the K reserves (Table 5).

The K deficit could be managed using the nutrient buildup and maintenance concept based on nutrient balance sheets to avoid long-term soil K depletion below optimum soil test K. While soil test K threshold may depend on the chemistry of pH-dependent charges (Levy et al., 1988; Melo et al., 2002) and the yield level (Parent et al., 2012b), the optimum soil test K of 1.6 mmol_c_ K dm^−3^ commonly used in Brazil for SLAN diagnosis appeared to be too high because no K shortage was found in the guava diagnostic leaf despite cumulated K deficits. To avoid excessive K deficits and excess of other nutrients on the long run in this guava agroecosystem, the parsimonious dosage of guava waste (e.g., 9 Mg ha^−1^ on dry mass basis) could be supplemented by mineral K fertilizers to maintain soil test K above 0.7 mmol_c_ K dm^−3^ at productivity levels of 60–61 Mg ha^−1^ or 1.0–1.2 mmol_c_ K dm^−3^ at productivity levels of 68–77 Mg ha^−1^ (Parent et al., 2012b). Soil test P appeared to be adequate at 10 mg P dm^−3^, as shown by excessive P supply to the leaf in TP specimens, indicating that the optimum soil test P range of 13–30 mg P dm^−3^ commonly used in Brazil was also too high. Nevertheless, being not compositional, the SLAN, and BCSR interpretation methods used in this paper to interpret the results of soil analysis could be revisited using compositional methods.

### Nutrient balances

Data mining and compositional data analysis techniques were used to estimate nutrient concentration ranges at high yield level. The balance concept provided a data transformation technique that reduced D parts to D - 1 orthogonal variables that accounted for nutrient interactions hidden in concentration values likely affecting concentration ranges (Bates, 1971). The accuracy of the knn classification across ilr values was 0.93, higher than 80% or more obtained in other studies using balances (Marchand et al., 2013; Parent et al., 2013a,c; Parent et al., 2015; Modesto et al., 2014) and up to 73% obtained in DRIS studies (Wadt et al., 2016).

Concentration ranges obtained from 100 000 Monte Carlo simulations can be compared to the literature. Natale et al. (2002) proposed ranges of 20–23 g N kg^−1^, 1.4–1.8 g P kg^−1^, 12–17 g K kg^−1^, 7–11 g Ca kg^−1^, 3.4–4.0 g Mg kg^−1^, 2.5–3.5 g S kg^−1^, and 20–25 mg B kg^−1^. Maia et al. (2007) suggested the following ranges: 20.2–25.3 g N kg^−1^, 1.4–1.5 g P kg^−1^, 19.0–21.7 g K kg^−1^, 7.7–8.3 g Ca kg^−1^, and 2.7–2.8 g Mg kg^−1^. Brazilian nutrient ranges thus appeared too high for N and Mg and too wide for K at lower bound for this guava agroecosystem. Different upper bound concentration values were also found for S and B. The N/P (Redfield) ratio ranged from 11.6 and 12.2 for TN specimens and 10.4–11.3 for TP specimens due apparently to excessive P levels in TP specimens. The lower critical value for the N/P ratio was 11.45. Other estimates of N/P ranges for guava at high yield level in Brazil were 11.5–13.1 (Parent et al., 2012a), 11.0–16.4 (Natale et al., 2002), 13.5–18.2 (Maia et al., 2007), and 10.3–13.7 (Hernandes et al., 2012), within the wide range of 10–20 as reported by Güsewell (2004). Hence, the soil test P of 10 mg P dm^−3^ appeared to be adequate to sustain the productivity of this guava orchard.

## Conclusion

Fertilization treatments with guava waste produced large cumulated K deficits during the 6 years of experimentation. However, the leaf and fruit tissues did not show any K shortage despite soil K mining. Guava waste could thus be recycled in guava orchard at parsimonious dosage to avoid N and P excess on the long run and supplemented with K to avoid K deficiency. On the other hand, depending on subtle climate change, imbalanced fertilization with guava waste affected to different degrees the nutrient budgets of the agroecosystem, the nutrient balances in fruit and foliar tissues, and fruit yield, and quality. Foliar nutrient balances could be monitored and diagnosed accurately against standards developed using tools of data mining and compositional data analysis.

Brazilian guava growers could benefit from this research by (1) revisiting optimum soil test K and P thresholds as well as the SLAN and BCSR interpretation models used in Brazil to achieve parsimonious nutrient management in guava agroecosystems where guava waste is recycled, and (2) monitoring the nutrient balance of fruit and foliar tissues to reach balanced fertilization in relation with targeted fruit yield and quality standards, and variations in climate conditions.

## Author contributions

HS: Field work, data collection and acquisition, statistical analyses and interpretation, literature review. SP: Data modeling (data mining, compositional data analysis, statistical analyses), graphics and tables, co-writer of the paper. DR, DA, VM: Field work, data collection, statistical analyses, and data set organization. WN: Conception and design of the experiment, laboratory methods, relation with the industrial partner, data interpretation, paper review, literature review. LP: Data set, literature review, co-writer of the paper.

## Funding

We are grateful to Fundação de Amparo a Pesquisa do Estado de São Paulo (FAPESP), Conselho Nacional de Desenvolvimento Cientifico e Tecnológico (CNPq), Indústria de Polpas e Conservas VAL Ltda., and the Natural Sciences and Engineering Research Council of Canada (NSERC-DG 2254) for financial support.

### Conflict of interest statement

The authors declare that the research was conducted in the absence of any commercial or financial relationships that could be construed as a potential conflict of interest.

## Abbreviations

BCSR: basic cation saturation ratio
CEC: cation exchange capacity
DGW: dry guava waste
DRIS: diagnosis and recommendation integrated system
FN: false negative
FP: false positive
ilr: isometric log ratio
knn: k nearest neighbors
RM: recommended mineral fertilization
SF: fresh guava waste
SBP: sequential binary partition
SLAN: sufficiency level of available nutrient
TN: true negative
TP: true positive.
